# Nasopharyngeal Swabs vs. Nasal Aspirates for Respiratory Virus Detection: A Systematic Review

**DOI:** 10.3390/pathogens10111515

**Published:** 2021-11-20

**Authors:** Matthew F. Flynn, Martin Kelly, James S. G. Dooley

**Affiliations:** 1 School of Biomedical Sciences, Ulster University, Cromore Road, Coleraine BT52 1SA, UK; jsg.dooley@ulster.ac.uk; 2 Altnagelvin Area Hospital, Glenshane Road, Londonderry BT47 6SB, UK; martin.kelly@westerntrust.hscni.net

**Keywords:** respiratory, virus, bacteria, nasopharyngeal, microbiome, infection

## Abstract

Nasal pathogen detection sensitivities can be as low as 70% despite advances in molecular diagnostics. This may be linked to the choice of sampling method. A diagnostic test accuracy review for sensitivity was undertaken to compare sensitivity of swabbing to the nasopharynx and extracting nasal aspirates, using the PRISMA protocol, Cochrane rapid review methodology, and QUADAS-2 risk of bias tools, with meta-analysis of included studies. Sensitivities were calculated by a consensus standard of positivity by either method as the ‘gold standard.’ Insufficient sampling methodology, cross sectional study designs, and studies pooling samples across anatomical sites were excluded. Of 13 subsequently eligible studies, 8 had ‘high’ risk of bias, and 5 had ‘high’ applicability concerns. There were no statistical differences in overall sensitivities between collection methods for eight different viruses, and this did not differ with use of PCR, immunofluorescence, or culture. In one study alone, Influenza H1N1(2009) favored nasopharyngeal swabs, with aspirates having 93.3% of the sensitivity of swabs (*p* > 0.001). Similarly equivocal sensitivities were noted in reports detecting bacteria. The chain of sampling, from anatomical site to laboratory results, features different potential foci along which sensitivity may be lost. A fair body of evidence exists that use of a different sampling method will not yield more respiratory pathogens.

## 1. Introduction

Accurate laboratory-confirmed diagnoses aid both timely treatment and surveillance of respiratory infections and are facilitated by rapid detection methods [1,2]. The frustration of false negative results for specific pathogen carriage experienced by clinicians, thus escalating treatment upon clinical suspicion alone, predates SARS-CoV-2 and leads to reliance on repeat tests and imaging [3,4]. Pathogens may be lost at sampling, transport, or laboratory processing. Suboptimal sensitivity has persisted for viruses, despite the adoption of polymerase chain reaction (PCR) as the gold standard above viral culture and direct immunofluorescence (DIF). PCR may be perceived as less sensitive in head-to-head comparison with DIF. However, due to its increased accuracy and the elimination of false positives potentially found in DIF, PCR avoids the decreased sensitivities in patients over 5 years of age seen with DIF [5,6,7]. 

**Figure 1:**
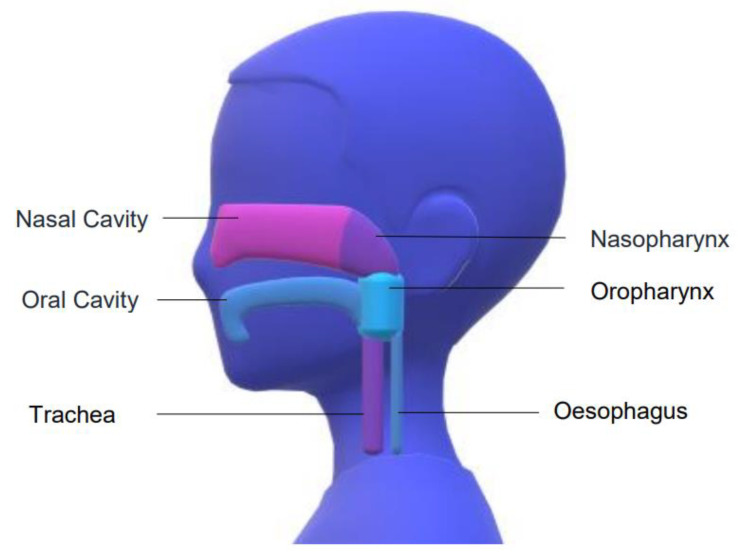
Anatomy of the Upper Respiratory Tract.

Higher viral loads present in the early course of a viral infection predominate more in the nose than in the throat, and slightly predominate in the nasopharynx than the anterior nasal cavity [8,9,10]. The nasopharynx is the uppermost portion of the throat lying at the back of the nasal cavity and accessible horizontally along the nasal floor, past occasionally obstructing turbinates and deviations of the septum (Figure 1). 

There is a related but distinct microbiome between the anterior nares (AN) and nasopharynx [11]. A nasopharyngeal swab (NPS) is inserted to a depth equal to the distance from the nostril to the earlobe (nasotragal length, NTL), or until the nasopharynx is felt (a depth of up to 14 cm), with less deep swabs ≥ 5 cm sampling the middle turbinate or AN [12,13,14]. The NTL in children and infants is shorter but remains considerably longer than the 2cm depth occasionally cited in NPS studies, as described in pediatric intubation [15]. Combined oropharyngeal and anterior nasal swabs are shown to be comparable in sensitivity to a single sample of the nasopharynx, whilst benefiting from higher patient satisfaction [16,17]. These combined throat/nose swabs have become recommended practice for self-administration of the test. Paired oropharyngeal/NPSs convey increased sensitivity compared to NPS alone [18]. The swab type used is an important consideration, with greater yield of respiratory epithelial cells and greater patient satisfaction with a flocked swab (akin to a miniature toilet brush) than a rayon-tipped swab (resembling a long cotton “ear” bud), but pathogen detection rate is equivocal [19,20]. Other important pre-laboratory variables such as pre-impregnation of swabs with transport media, immediate placing in medium following collection, or refrigeration of the sample appear to add little to the diagnostic yield [21,22]. Nasal aspiration (NA) involves suction of mucous from the nasal cavity in a mucous trap subsequently rinsed with saline or transport medium. The similar but distinct nasal wash (NW), similarly described as the Naclerio method, is obtained by the drainage without suction of a small volume of saline flushed into the nose [23,24]. Samples obtained by nose blowing are not widely used, and the high prevalence of Staphylococcus aureus in these samples suggests microbial contamination from the AN, or external skin [25,26]. Unsurprisingly, a review of methods for Influenza detection found increased yield when pairing combinations of diverse methods [27].

In the absence of an unrelated gold standard, a composite reference standard combining these two imperfect tests can be used to create a “consensus standard” or “positive” rule against which to compare sensitivities [28,29]. In such cases without a true reference standard against which to expose false negatives, overall sensitivity will be overestimated. Nevertheless, head-to-head comparison of two such techniques with eyes open to this overall underestimation of total pathogen presence will still yield the best from two relatively “bad” options [30].

The current true sensitivity of nasopharyngeal swabbing for SARS-CoV-2 is estimated at 71–98% [31]. An increase in test sensitivity from 70–90% is enough to decrease by more than half the pre-test probability of infection at which one would still suspect an infection despite a negative test [32]. Thus, even a small increase in sensitivity by alteration of method could alter clinical practice. This systematic review was designed to compare the swabbing and aspirates per pathogen, laboratory method, and collection method using systematic methods.

## 2. Results

**Figure 2:**
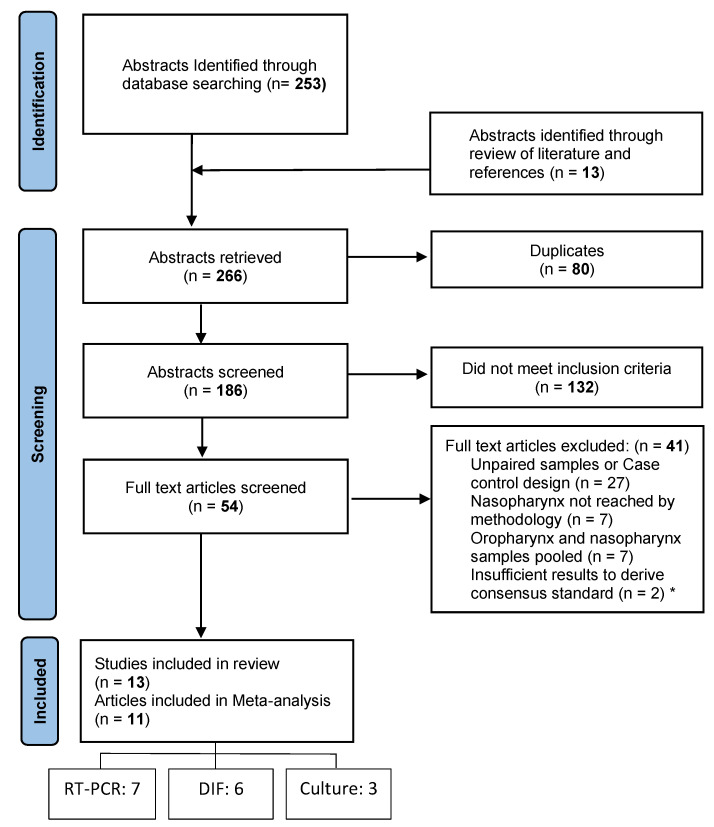
Preferred Reporting Items for Systematic Reviews and Meta-Analyses Flow chart. RT PCR: Reverse Transcriptase Polymerase Chain Reaction; DIF: Immunoflourescence; * Several studies met more than one exclusion criteria and/or used multiple laboratory methods.

The initial search identified 253 articles (Figure 2). A further 13 were added from searching reference lists of key papers and reviews. After screening titles and abstracts, the abstracts of 186 publications were screened. Of the 54 abstracts that met the eligibility criteria, only 13 were eligible to be included in the final review after the full texts were reviewed. One study was an abstract published within a conference proceedings supplement.

### 2.1. Risk of Bias

**Figure 3:**
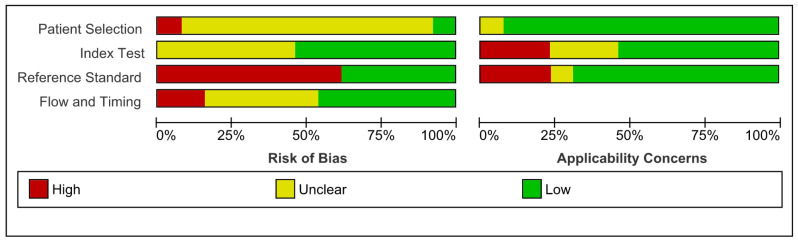
Methodological Quality Summary Chart.

**Figure 4:**
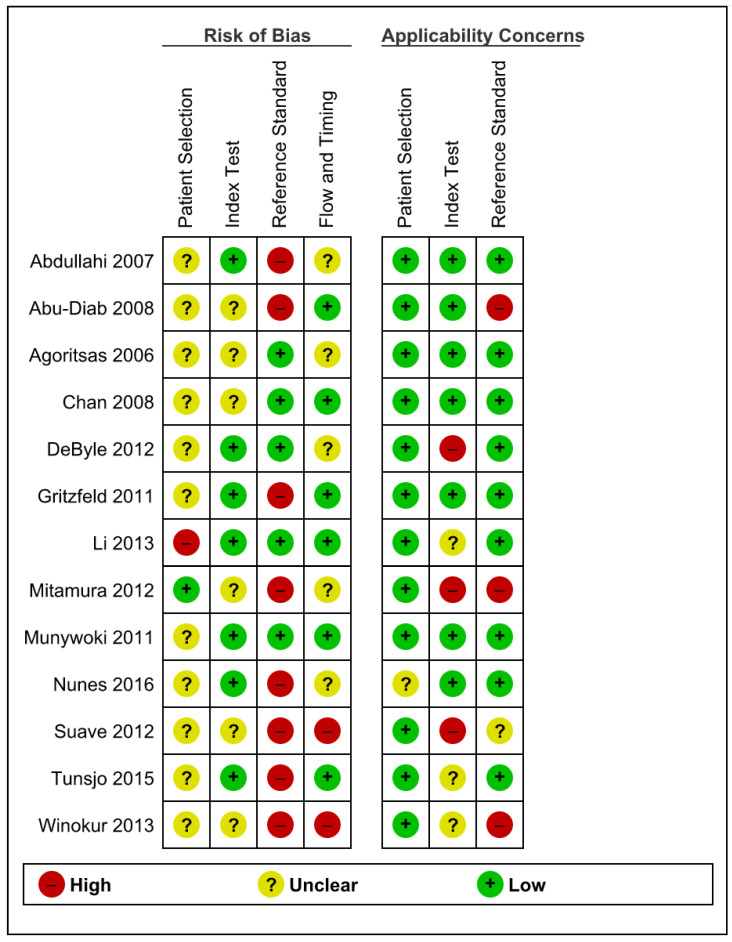
Methodological Quality Summary Table.

Using the QUADAS-2 tool, overall risk of bias was high: 8/13 studies displayed “high” risk of bias. Applicability concerns were “low” in 8/13 studies (Figure 3 and Figure 4). Lack of information on the patient selection process in 11/13 led to “unclear” risk of bias under patient selection. There were “low” applicability concerns due to patient selection, but this was a reflection of the review question including any and all populations; there was considerable homogeneity of age, ethnicity, or disease status. Three studies declared material support from companies manufacturing the testing kits. 

### 2.2. Heterogeneity

The available literature was complicated by heterogeneity of participant age, participant health, laboratory methods, and collection methods, even within collection methods given the same name (Table 1). 

### 2.3. Laboratory Methods

Methods used to detect pathogen carriage varied across studies. Eleven species-specific molecular methods were employed: seven using PCR and six using direct immunofluorescence, including one using both. Five different immunoassay kits were used for immunofluorescence. Three were cultured on inoculated Skim-milk-tryptone-glucose-glycerin for bacteria and one used the “R-mix” rapid culture method for viral growth. Where recorded, a negative cut-off was assigned to the Ct value > 40, with the exception of one, which was >35. Six used saline to transport the samples, six used viral transport medium, one used Guanidinium Thiocyanate Buffer, and one was unspecified. 

### 2.4. Virology

**Figure 5:**
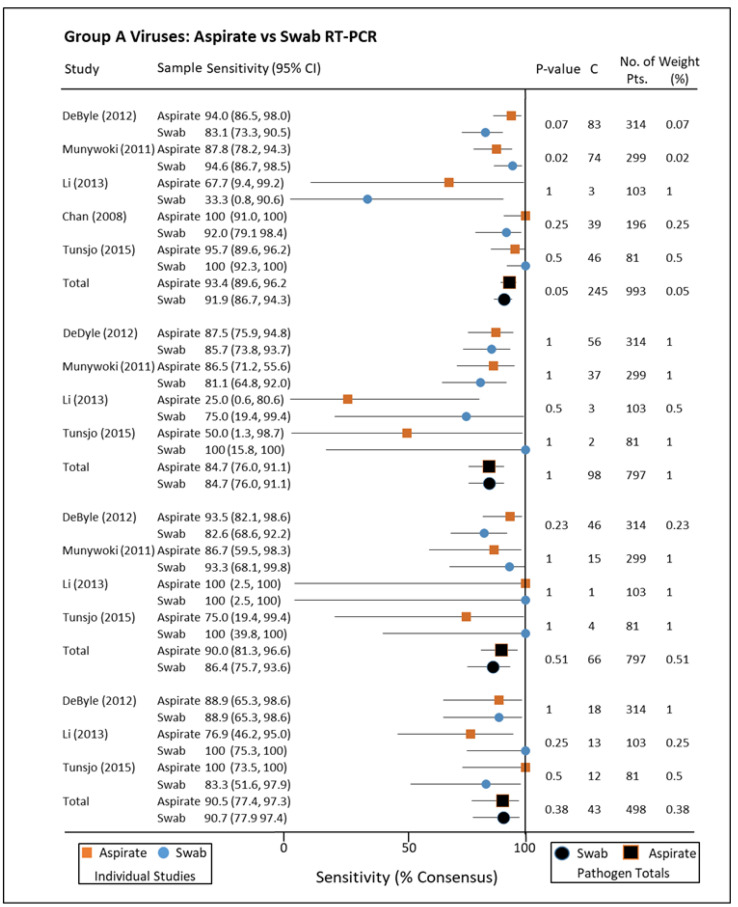
Group A viruses: aspirates vs. swab, reverse transcriptase polymerase chain reaction. RSV: respiratory syncytial virus; CI: confidence interval; C+: total consensus positive; No. of Pts.: number of patients; RT-PCR: reverse transcriptase polymerase chain reaction.

**Figure 6:**
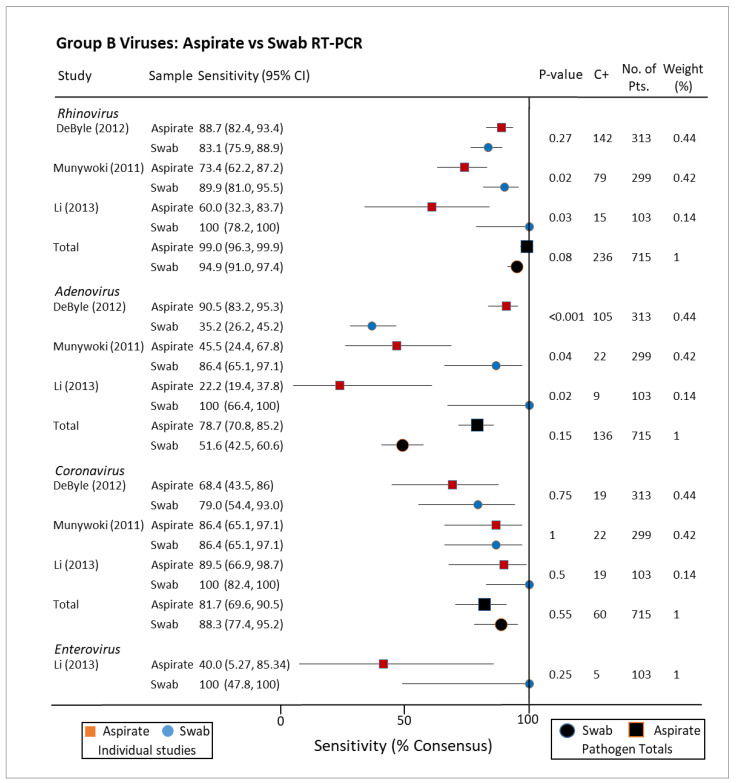
Group B viruses: aspirates vs. swab, reverse transcriptase polymerase chain reaction. CI: confidence interval; C+: total consensus positive; No. of Pts.: number of patients; RT-PCR: reverse transcriptase polymerase chain reaction.

**Figure 7:**
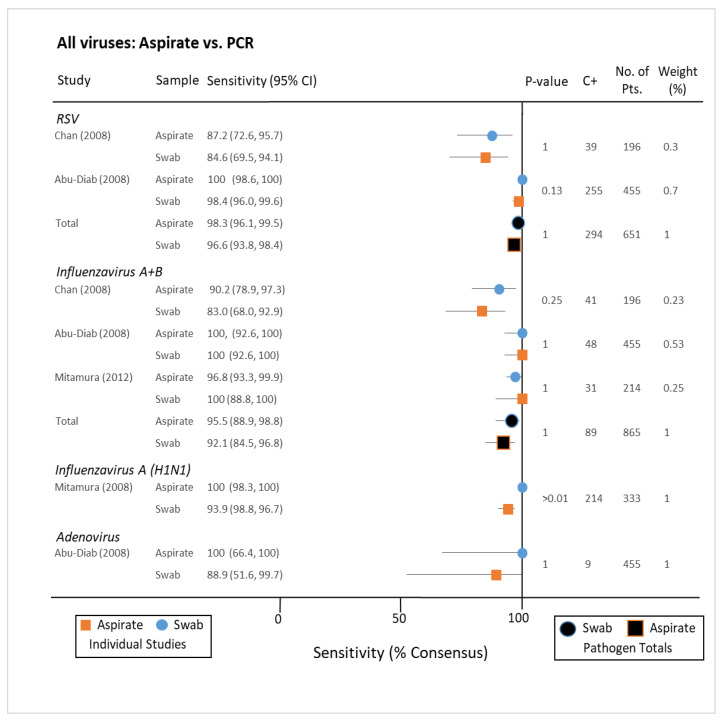
All viruses: aspirates vs. swab, direct immunoflourescence. RSV: respiratory syncytial virus; CI: confidence interval; C+: total consensus positive; No. of Pts.: number of patients; DIF: direct immunofluorescence.

PCR analysis of NA and NPS for group A pathogens, i.e., those associated with hospitalising illness, included respiratory syncytial virus (RSV), parainfluenza virus, metapneumovirus, and influenza A+B (Figure 5). Sensitivities as a fraction of consensus standard (positive for either collection method) ranged between 84% and 96% for these pathogens by both collection methods. A similar lack of statistical dissimilarity was found when stratifying for group B viruses Rhinovirus, Adenovirus, Coronavirus, and Enterovirus (not normally associated with severe disease), but the range of sensitivities was greater (Figure 6) [33,34,35,36,37]. When immunofluorescence was utilized for diagnosis, there was little difference in sensitivity for either NA or NPS with the exception of one study exclusively testing for Influenza A H1N1(2009) (*p* < 0.001) [38,39]. When compared to nasal washes (similar to aspirates but without the use of suction), nasal swabs for influenza were similarly demonstrated to have greater sensitivity for detecting Influenza in one study of 122 participants (Figure 7) [40]. Another study of 89 paired samples, 25 nasopharyngeal washes compared to 26 swabs showed RSV carriage (*p*-value = 1) [41]. Many studies described findings seeming to advantage one collection method or other as a standalone, but this disappeared when pooled with others testing for the same pathogen by the same techniques. Indeed, when combined, the sensitivities of both collection methods for Parainfluenza virus were the same (84.7%/84.7%). 

### 2.5. Bacteriology

Neither aspirate-wash versus swab for *Bordetella pertussis* PCR nor non-typable *Haemophilus influenzae* in culture yielded a significant advantage [42,43]. Collated sensitivities of the Naclerio method vs. NPS for a variety of species in 24 healthy British adults favored NPS for *Neisseria* (60.2%/100%), Diptherioids (66.7%/100%), and Alpha-haemolytic streptococci (18.8%/100%, *p* < 0.001), the Naclerio method for *Staphylococcus aureus* (100%/66.7%), and equivocal for *Moraxella catarrhallis* [33]. A similar number of Kenyan infants presenting to hospital with mild illness not requiring hospitalization, and having a suction catheter passed to the nasopharynx grew *Streptococcus pneumoniae* in 55 samples. In comparison, 47 (85.0%) of these grew the pathogen on their NPS (*p* = 0.005) [44]. These high yields may reflect the later adoption of the pneumococcal vaccine in Kenya in 2011 [45].

**Table 1:** Characteristics of included studies.

Author	Year	Patients	Wash/Aspirate	Population	Lab Technique	Swab
DeByle [33]	2012	314	aspirate-wash	infants	RT-PCR	flocked
Chan [35]	2008	196	aspirate	Infants, children	DIF and RT-PCR	unknown
Suave [41]	2012	89	wash	Infants, children	DIF	flocked
Munywoki [34]	2011	299	aspirate-wash	Infants, children	RT-PCR	flocked
Abu-Diab [39]	2008	455	aspirate	Infants, children	DIF	flocked
Agoritsas [40]	2006	122	wash	Infants, children	DIF and viral culture	foam
Tunsjo [37]	2015	81	aspirate	infants	RT-PCR	flocked
Nunes [42]	2016	484	aspirate-wash	infants	RT-PCR	flocked
Li [35]	2013	103	aspirate-wash	adults	RT-PCR	flocked
Abdullahi [44]	2007	62	aspirate-wash	infants	culture	rayon tipped
Winokur [43]	2013	15	wash	adults	DIF	flocked
Gritzfeld [24]	2011	24	wash	adults	culture and RT-PCR	rayon tipped
Mitamura [38]	2012	330	aspirate	children, adults	DIF	unknown

RT-PCR: reverse-transcriptase polymerase chain reaction; DIF: direct immunoflourescencel; aspirate-wash: studies that tested instilled saline removed by a suction catheter; aspirate: suctioning of nasal secretions without flushed water; wash: free drainage of instilled water.

## 3. Discussion

This systematic review found a moderate body of evidence comparing nasopharyngeal swabs with aspirates and washes with no significant difference in sensitivity. These are sufficient to recommend either method for optimal bacterial and viral coverage. Findings were predominantly from PCR-based diagnostics, comparing swabs with suction-using aspirates, and comparing viruses. Beyond these strata, data were sparse, particularly for purely wash-based methods, and for detection of bacteria. Statistical significance of higher NP swab sensitivity was high for H1N1(2009), but this same study found no clear advantage to this method for Influenza A or B. Some of the studies included were from an era of DIF and culture, which will have less relevance in future with the predominance of genomic diagnostics. Furthermore, the mechanics of removing pathogens from their in vivo habitat are poorly understood—swabbing and brushing are more abrasive and likely to access deeper layers of the mucosal barrier. Differences in adhesive properties of bacteria and viruses, as extra- and intracellular agents, respectively, remain, and the adhesive properties of biofilm also require further characterization [46]. 

### 3.1. Limitations of the Study

The abundance of confounding variables can only be accounted for in part by risk of bias assessments. Variations in transport, time in storage, and laboratory staff have not been considered. In the absence of a reference standard, the approximation of similar sensitivity rates for multiple different method comparisons implies saturation of this diagnostic chain; only on the smaller studies were large differences in sensitivities seen, and these not only disappeared when pooling studies but when comparing with better powered studies. This implies a limiting common denominator to all. Along the diagnostic chain from mucosa to laboratory bench, the step least likely to be controlled is the specific anatomical sampling technique. Single operator sampling under direct vision and controlled conditions, as described elsewhere, would be required for complete confidence in sampling [47]. This methodological heterogeneity can be controlled for in part by case matched control studies, where such variations were likely to affect both groups equally. 

The Cochrane rapid review protocol proved a portable and efficient mode of prompt evidence synthesis for this timely clinical question with the use of open access freeware. Rapid reviews maintain a moderate degree of quality assessment while removing full search saturation and streamlining study selection and data extraction. As rapid reviews are an evolving methodology, it is unclear the extent to which methodological omissions compromise the quality of these results [48].

### 3.2. Limitations of Current Literature 

Most published works to date focus on viral detection only. The clinical application of viral detection is not straightforward. Such techniques indicate only pathogen carriage and not severity of respiratory disease. Variations in viral shedding in the upper respiratory tract include: a shorter time to peak viral concentrations in saliva in SARS-CoV-2 compared to severe acute respiratory syndrome (SARS) (5 days vs. 7–10 days) and completed viral shedding of Influenza virus in adults is only completed around 5-7 days compared to infectivity persisting beyond 10 days in infants [49,50]. The attributable fraction, namely the percentage of times a disease is caused by a detected virus, ranges from 12% for Rhinovirus to 93% for RSV [51]. Thus, even truly reliable results do not confirm disease. Ergonomics also merit consideration: the washing method has been described as more comfortable for adults than a nasopharyngeal swab, and in children, anterior nasal swabbing results in lower infant distress score than an aspirate [23,31]. The Naclerio method requires a degree of coordination; however, that restricts its use to adults. The perceived and achieved discomfort may also be presumed to affect the thoroughness and accuracy in a linear fashion. A need for better understanding of anatomy of the nose in the literature is also called for. As the NP cannot be reached except via contact with the turbinates and septum, nasopharyngeal swabs may be more appropriately named a “pan-nasal” swab.

### 3.3. Future Directions 

How else to augment sensitivity? Moist swabs appear to add little advantage over dry swabs; repeated titres in severe cases may give a retrospective estimate of the sensitivity of initial samples [52,53]. This study looked at viral and bacterial carriage. This still leaves the issue of the best method for sampling wider nasal ecology in the era of next generation sequencing. Increased diversity of microbiota can be removed from brushing the inferior turbinate compared with nasal washing [54]. Given this niche-specific diversity, it is difficult to assess if such variations denote a different topographical area being sampled or a different constellation of organisms being easier to remove from the nasal lining. Gradual mapping of the nasopharyngeal microbiome over lifespan is a prerequisite to the future application of such technologies to diagnostics and therapy [55]. Emerging point-of-care diagnostics are in readiness to accelerate all accurate and reliable respiratory pathogen sampling to guide timely treatment and surveillance on a global level but will rely on the most sensitive sampling methods available [56].

## 4. Materials and Methods

### 4.1. Protocol

The protocol followed guidance from the Cochrane Rapid Reviews Methods Group. Rapid reviews streamline evidence synthesis methods such as one-person title screening and elimination of grey literature, while maintaining a high degree of rigor [57]. The preferred reporting items for systematic reviews and meta-analyses (PRISMA) and the Cochrane handbook for diagnostic test accuracy reviews were used [58,59]. The protocol was registered with the International Prospective Register of Systematic Reviews (PROSPERO) prior to formal screening of search results against eligibility criteria. Consultation with stakeholders in medicine and a public focus group aided the study design.

### 4.2. Inclusion Criteria

All studies comparing sensitivity in microbiological sampling for the upper respiratory tract were sought. Variations in viruses, bacteria and fungi detected and differing laboratory techniques were included and stratified by these categories in the reported results. Studies were limited to those in humans, and those since the first publication of WHO guidelines on swab and aspirate collection in 2006 [32]. Pooled samples between different anatomical sites were excluded, such as swabbing of the nose and throat by the same swab or where samples were not paired from the same patient. Where a consensus standard was not included or this information could not be calculated, these studies were also excluded. 

### 4.3. Nasopharynx Definition

Nasopharyngeal swabs were defined as swabbing to a depth of >5 cm in adults and >3 cm in children or with relevant reference to surface anatomy, by citing WHO guidelines for swab collection, or following the same methodology as other studies to the same effect. Where these data were lacking, the authors were contacted for clarification, and/or judgments made on appropriate anatomical nomenclature and documented staff training. Broad interpretation of the term “nasopharynx” elsewhere in the literature to include the middle turbinate and AN led to the inclusion of ‘nasopharyngeal’ studies without further methodological detail but graded ‘high’ for applicability concerns.

### 4.4. Search Strategy

Cochrane CENTRAL, MEDLINE, and Embase were searched on 09/06/2020, followed by supplemental exploration of reference lists of review articles. Help of a specialist health librarian was sought to ensure that wording variations and the correct Boolean operators optimized search saturation. Full details of search terms are accessible online via the protocol (PROSPERO registration no. CRD42020189577).

### 4.5. Screening

A title and abstract screening form was piloted using 30 abstracts and adopted without modification to dual screen 20% of abstracts with conflict resolution. Remaining abstracts were screened by one reviewer, and a second reviewer screened all excluded abstracts. A full text screening form was piloted using 5 full text articles with the same process. Rayyan QCRI was used to streamline the selection process. 

### 4.6. Data Extraction

Results were stratified by virus, bacteria, and differing laboratory techniques. Aspirates are defined as extraction of fluids by suction catheter and washes as free drainage of instilled saline into a dish. A third hybrid method, where flushed water was then aspirated, has been incorporated under Aspirates [32]. Where studies included multiple sampling methods, only relevant data were used. Where the consensus standard was equal to the sensitivity of one of the sampling methods, i.e., there were no false negatives for one of the two collection methods, these data were acknowledged as having a ‘high’ risk of bias. 

### 4.7. Data Analysis

Meta-analysis was summarized for studies with similar methodologies; for more sparse and/or heterogeneous evidence, a narrative summary is offered. Sensitivity analysis and McNemars’ test for paired samples were derived using Medcalc and Scistat online statistical software, respectively. 

### 4.8. Risk of Bias Assessment 

The QUADAS-2 risk of bias tool for diagnostic test accuracy reviews was used to grade risk of bias and applicability concerns by one reviewer (MF) with verification by second viewer (JD). Due to risks unique to this study, the questions “Were false negatives two-sided?” under reference standard, and “Were separate nostrils used?” were added under flow and timing. Risk of bias tables were generated in Review Manager 5.

## Data Availability

Full protocol data, including inclusion and exclusion criteria and search terms can be accessed on the PROSPERO register registration no. CRD42020189577 https://www.crd.york.ac.uk/prospero/display_record.php?ID=CRD42020189577 (accessed on 10 October 2021); Web and mobile application Rayyan QCRI can be accessed for free at https://www.rayyan.ai/ (accessed on 10 October 2021); Diagnostic test evaluation calculator (MedCalc) can be accessed at https://www.medcalc.org/calc/diagnostic_test.php (accessed on 10 October 2021); McNemar’s test on paired proportions (SciStat) can be accessed at https://www.scistat.com/statisticaltests/mcnemar.php (accessed on 10 October 2021); Review Manager 5 (The Cochrane Collaboration) can be accessed at revman.cochrane.org (accessed on 10 October 2021); QUADAS-2 Risk of Bias tool (University of Bristol) can be accessed at https://www.bristol.ac.uk/population-health-sciences/projects/quadas/quadas-2/ (accessed on 10 October 2021).

## Abbreviations

PRISMA	Preferred Reporting Items for Systematic Reviews and Meta-Analyse
QUADAS-2	Quality Assessment of Diagnostic Accuracy Studies-2
(RT-)(q-)PCR	(Reverse Transcriptase) (quantitative)Polymerase Chain Reaction
SARS(-CoV-19)	Severe Acute Respiratory Syndrome (Coronavirus-2019)
DIF	Direct Immunofluorescence
NPS	Nasopharyngeal Swab
NTL	Naso-tragal Length
NA	Nasal Aspiration
NW	Nasal Wash
PROSPERO	International prospective register of systematic reviews
WHO	World Health Organisation
Rayyan QCRI	Rayyan Qatar Computing Research Institute
RevMan	Review Manager
16S-rRNA	16S-Ribosomal Ribonucleic Acid
